# A Replicative Self-Renewal Model for Long-Lived Plasma Cells: Questioning Irreversible Cell Cycle Exit

**DOI:** 10.3389/fimmu.2013.00460

**Published:** 2013-12-18

**Authors:** Reuben M. Tooze

**Affiliations:** ^1^Section of Experimental Haematology, Leeds Institute of Cancer and Pathology, University of Leeds, Leeds, UK; ^2^Haematological Malignancy Diagnostic Service, Leeds Teaching Hospitals NHS Trust, Leeds, UK

**Keywords:** plasma cell, cell cycle, self-renewal, quiescence, myeloma, monoclonal gammopathy, gene expression, lifespan

## Abstract

Plasma cells are heterogenous in terms of their origins, secretory products, and lifespan. A current paradigm is that cell cycle exit in plasma cell differentiation is irreversible, following a pattern familiar in short-lived effector populations in other hemopoietic lineages. This paradigm no doubt holds true for many plasma cells whose lifespan can be measured in days following the completion of differentiation. Whether this holds true for long-lived bone marrow plasma cells that are potentially maintained for the lifespan of the organism is less apparent. Added to this the mechanisms that establish and maintain cell cycle quiescence in plasma cells are incompletely defined. Gene expression profiling indicates that in the transition of human plasmablasts to long-lived plasma cells a range of cell cycle regulators are induced in a pattern that suggests a quiescence program with potential for cell cycle re-entry. Here a model of relative quiescence with the potential for replicative self-renewal amongst long-lived plasma cells is explored. The implications of such a mechanism would be diverse, and the argument is made here that current evidence is not sufficiently strong that the possibility should be disregarded.

## Introduction

Entry into cell cycle quiescence accompanies the final phenotypic maturation of plasma cells (1). In short-lived plasma cells the maintenance of quiescence is rendered irrelevant by cell death. In contrast long-lived plasma cells may potentially survive for the lifetime of the organism (2, 3), and the mechanisms maintaining quiescence in such populations are of much greater significance. The current paradigm holds that cell cycle exit is permanent and irreversible for all plasma cells (2, 4, 5). However it may be timely to re-evaluate this paradigm and consider an alternate possibility: that long-lived “memory” plasma cells adopt a quiescent state, controlled by their niche environment, but maintain the potential for replicative self-renewal through cell division. The implications of such a mechanism are diverse, and the evidence against is potentially not sufficiently strong that the possibility should be disregarded. Recent progress in generating and maintaining long-lived human plasma cells *in vitro* may help to address some aspects of this issue.

## Discussion

### Lifespan of plasma cells

The nature of plasma cell lifespan and the concept of irreversible cell cycle exit accompanying terminal differentiation are intertwined. Early observations of potential plasma cell longevity were largely set aside in favor of the view of continuous generation of short-lived plasma cells (6, 7); and in the context of cell cycle exit coupled to functional differentiation, and imminent cell death the concept of irreversible cell cycle exit is natural and follows the prevailing pattern in other short-lived hemopoietic effectors.

The critical transition in our understanding of plasma cell longevity came with the studies of Manz et al. (8) and Slifka et al. (7), whose works combined to provide proof of the existence of long-lived plasma cells, which preferentially resided in the bone marrow and made a central contribution to long-term humoral immunity. Subsequent work from other labs in mouse models has also pointed to extended lifespans, although the half-life predictions vary somewhat with the type of assay and vaccination strategy employed, and in recent data include dynamic changes in long-lived plasma cells in response to systemic inflammation (5, 9, 10). In earlier continuous tritiated-thymidine incorporation studies in rat, antibody-secreting cells in the bone marrow showed more general labeling reaching near 40% by 10 days (11), but it has been argued that these experiments may have overlooked long-lived quiescent plasma cells since antigen-specific populations were not assessed (12).

Serological studies in human combined with the persistence of plasma cells after therapeutic B-cell depletion point to significant lifespans for human bone marrow plasma cells (13). While direct evidence of plasma cell longevity in man is limited, *in vitro* generated human plasma cells can certainly persist as non-dividing cells for months (14). A view of the bone marrow plasma cell compartment, encompassing the decay of antibody titers after therapeutic B-cell depletion, would include a heterogenous mix of plasma cell populations, many with relatively short half-lives in the region of <100 days, as well as populations of longer-lived cells persisting well beyond this time-frame. In human bone marrow such heterogeneity is potentially reflected in phenotypic differences in bone marrow plasma cells (G. Arumugakani and A. Rawstron, personal communication).

### Differentiation and the permanence of cell cycle exit

While the shift toward a general acceptance of long-lived bone marrow plasma cell has occurred, the paradigm that all plasma cells have irrevocably exited cell cycle has remained (2, 4, 5). Terminal differentiation as a concept encompasses the acquisition of high functional specialization and the loss of potential for alternate cell fates. This is frequently linked to irrevocable cell cycle exit. This clearly pertains in the context of short-lived effector cells that die soon after completing differentiation and exiting cell cycle. In contrast in long-lived cells functional specialization is not necessarily linked to irrevocable cell cycle exit (15–19). Schwann cells provide a well-studied example of cells with high functional specialization that enter a quiescent rather than post-mitotic state, and can re-enter cell cycle in response to injury or growth factor stimulation (18). However the ability of differentiated cell populations to re-enter cell cycle also extends to other systems traditionally viewed as terminally differentiated such as cardiac myocytes (15, 16, 19). Recently such concepts have also been extended to tissue resident macrophage populations (20–22). Given that such examples exist in other tissues with complex organization, and in immunological populations conventionally viewed as deriving from hemopoietic repopulation, it is reasonable to re-evaluate the paradigm that all differentiated plasma cells have necessarily exited cell cycle in an irrevocable fashion.

While there is little evidence directly in support of long-lived plasma cells undergoing self-renewal by cell division there is also relatively scant data in direct opposition. Several early studies demonstrated that plasma cells but not their proliferating precursors were resistant to hydroxyurea in short-term cultures (23–25). This is consistent with cell cycle exit, but does not distinguish the nature of this quiescence. Phenotypically mature human plasma cells generally lack Ki67 expression by flow cytometry (26), but low levels of proliferating plasma cells are detectable (27), equally flow cytometry only identifies a small fraction of hemopoietic stem cells (HSCs) as detectably in cell cycle (28). The need for long-lived plasma cell renewal may be different in organisms with extended lifespans and longer intervals to sexual maturity, but in the murine studies a slight accumulation of BrdU-labeled antigen-specific plasma cells over time appears to be present in the continuous feeding arm of the original Manz et al. study (8). Additionally an interesting recent study examined pulsed BrdU incorporation into bone marrow plasma cells under a range of immunization scenarios (5). At 60 and 100 days after primary immunization a very low, but above background, level of BrdU incorporation into antigen-specific plasma cells appears to be present. While such data can be interpreted in the context of the prevailing paradigm as newly formed plasma cells arising from B-cell differentiation, it can also be argued that the cell of origin from which a BrdU-labeled plasma cell derives is not known in such studies, particularly after an extended period post immunization. Some or all of the BrdU labeling could derive from incorporation into pre-existing plasma cells that arose following the initial immunization.

### Cellular relationships, the niche, and cell cycle

Under a range of experimental conditions the process of cell cycle exit during plasma cell differentiation occurs in an orderly fashion (14, 29–31), suggesting that the core mechanism is a common feature shared by all plasma cells regardless of cell of origin or activating stimulus. Whether such consistency pertains to the maintenance of cell cycle exit in differentiated plasma cells is less certain. The ability of cellular environment to influence plasma cell biology is a central theme of long-term plasma cell survival and the bone marrow plasma cell niche is critical to the persistence of these cells (32, 33). This niche is in part defined by stromal cells that also support multipotent hemopoietic precursors (34). In addition to stromal cells many more fluctuant hemopoietic populations have been implicated as contributing factors to plasma cell survival (34–38). This complexity has been summarized in the concept of the multicomponent plasma cell niche (39).

For the principle other long-lived quiescent bone marrow population, the HSC, a remarkably close relationship exists between the niche environment and quiescent state, with a range of niche factors implicated in maintaining quiescence (40–44). Using HSCs as an example, if replicative self-renewal can occur in plasma cells then it is likely that triggers will include changes in niche factors that link quiescence to survival, and physiologic signals that promote transient proliferation. If both quiescence and survival are directly linked to niche occupancy, then the partial displacement of a long-lived plasma cell from its niche, or the depletion of limiting niche factors by competing populations could represent a trigger for replicative self-renewal.

### Cell cycle in plasma cell differentiation

In general clonal expansion precedes plasma cell differentiation representing an intrinsic feature of an effective immune response. In model systems plasma cell differentiation is a process directly linked, at a mechanistic level, to cell division (23, 45–47). Indeed whether plasma cell differentiation can occur in the absence of cell division is uncertain. Elegant modeling approaches indicate that the fate adopted by differentiating B-cells is determined in a stochastic fashion linked to cell division (48).

While cell division is thus an essential component of the plasma cell differentiation program, this program does not complete if exit from cell cycle is prevented (1, 30). This is not a unique feature of plasma cell differentiation, but a general one of differentiation programs. Indeed much more is known of how cell cycle control intersects with transcriptional programs of cellular differentiation in other lineages (49). General features are that control of G1 progression and regulators of the RB/E2F pathway are central to these processes, but show lineage specific relationships. Furthermore cell cycle regulators can directly impact on transcriptional regulation and the execution of differentiation programs (49).

Direct experimental evidence indicating that cell cycle exit is necessary for plasma cell differentiation comes from analysis of p18INK4C, whose presence in differentiating B-cells is essential for plasma cell differentiation (30). p18INK4C (CDKN2C) is a repressor of the CyclinD-CDK4/6 complex and thus controls the initial phosphorylation of RB which is required for subsequent hyperphosphorylation by Cyclin-E-CDK complex and G1/S progression (1). CDKN2C is induced in parallel with BLIMP1 (PRDM1), the principle transcription factor defining the committed transition to plasma cell differentiation (50). CDKN2C deficient B-cells induced to undergo plasma cell differentiation express BLIMP1 and initiate the gene expression program of plasma cell differentiation, but fail to complete the process of differentiation appropriately, and never achieve the high secretory activity of terminally differentiated plasma cells (30). In a more recent analysis the same laboratory has extended these investigations to examine the transition of highly proliferative “intermediate plasma cells” to mature plasma cells, the former cell state being similar to plasmablasts. CDKN2C was progressively induced from activated B-cell to intermediate and then mature plasma cell state, while CDKN1B (p27KIP1) was selectively induced in mature plasma cell in which both CYCLIN-D2 and -E were also maintained (50). Failure to induce CDKN2C led to cell death amongst “intermediate plasma cells.” These data suggest that cell cycle inhibitors may act sequentially in the differentiation process, and indicate that apoptotic pathways provide a fail-safe controlling plasmablast that do not appropriately exit cell cycle.

A direct link between the differentiation program and cell cycle exit also lies in the ability of BLIMP1 to repress *MYC* (51, 52). BLIMP1 expression accumulates with time during plasma cell differentiation (53), and is well established as a critical regulator of this process necessary to mediate the genetic reprograming from B-cell to plasma cell (54, 55). BLIMP1 is implicated in the repression of multiple targets associated with cell proliferation (54), and can bind at the promoters of genes associated with kinetochore function (*SPC25* and *CENPH*) (56), which are also repressed at the plasmablast to plasma cell transition (14), providing an additional mechanism by which it may impact on cell division in plasma cells. Expression of BLIMP1 accumulates during differentiation, precedes the entry into cell cycle quiescence, and is abundantly expressed in mouse and human proliferating plasmablast populations (14, 31, 50, 53). BLIMP1 can recruit several different chromatin modifiers (57–59), which together provide the potential for either labile or stable epigenetic regulation. In a cumulative expression model the gradual increase of BLIMP1 may be linked to sequential and progressively more stable extinction of different gene expression programs. Under such a model the proliferative program would represent a late target repressed once BLIMP1 levels pass a particular threshold at the plasmablast stage. The inability of BLIMP1 to compensate for p18INK4C (50), suggests that the role of BLIMP1 in cell cycle control could lie on the one hand in attenuating the impetus for cell cycle progression and on the other in reinforcing the decision through epigenetic repression of proliferation genes. Nonetheless at present little is known of how the transcriptional program of plasma cell differentiation intersects with the machinery of cell cycle control and exit. While BLIMP1 acts as a regulator of terminal differentiation in both B- and T-cell lineages (55, 60, 61), this transcription factor can also play roles in cellular populations with stem cell properties (62–65). Thus BLIMP1 expression *per se* is not necessarily strictly linked to irreversible cell cycle exit.

### BLIMP1, BCL6, and myeloma plasticity

During B-cell differentiation BLIMP1 is involved in counter-regulatory relationships with PAX5 (66–68), SPIB (54, 69), and BCL6 (54, 70, 71). These regulatory interactions may be further modified by BACH2 dependent BLIMP1 repression (72–75). Together these interactions play critical roles in limiting and modulating PC differentiation. A notable feature of the “intermediate plasma cells” generated during murine splenic immune responses, was the apparent co-expression of BLIMP1 and BCL6 in a significant fraction of cells (9%), when analyzed at single cell level (50). However this co-expression was likely to represent a transient state as most cells expressed either factor in a mutually exclusive fashion. Interestingly, in a myeloma cell line model co-expression of BCL6 and MTA3 can drive a phenotypic reversion with B-cell antigen expression (76). Although evidence for BCL6 expression in primary myeloma is limited, some evidence of expression has been observed (77). While BCL6 expression and phenotypic reversion may provide an explanation for observations in advanced myeloma, and myeloma cell lines, it is important to distinguish between such transformed states and those operating in normal plasma cells, and early plasma cell neoplasms. In cell lines and advanced myeloma several co-operating oncogenic events contribute to extensive cell cycle deregulation (78, 79). In addition loss of epigenetic control, with a global increase in DNA hypo-methylation, is a feature of the monoclonal gammopathy of undetermined significance (MGUS) to myeloma transition (80). Cell proliferation itself imposes a burden on maintaining epigenetic regulation, requiring the re-establishment of appropriate control with each division (81). Thus rapid proliferation is likely to contribute to plasticity of gene expression in advanced myeloma, and may be intrinsically linked to reversion to an “intermediate plasma cell” or plasmablast-like state. Indeed in some instances patients present *de novo* with disease that blurs the boundaries between “plasmablastic myeloma” and “plasmablastic lymphoma,” leading to significant diagnostic and therapeutic questions (82). However, in normal plasma cells, and early neoplasia the balance is strongly in favor of maintaining appropriate epigenetic control and if cell cycle re-entry does occur, then limiting the frequency of this event. Thus, while consideration of phenotypic plasticity and its transcriptional control is relevant in advanced plasma cell malignancies, this must be distinguished from the control processes potentially operating in normal plasma cells, and early plasma cell malignancy under consideration here.

### Nuclear organization as a link to stable cell cycle exit

A classical feature of mature plasma cells is the organization of the nucleus such that dense chromatin condensation is observed in a ring like pattern at the nuclear periphery (83). This generates the “clock-face” nucleus of the mature plasma cell. The significance of this nuclear architecture is unknown, but compaction of heterochromatin can provide a mechanism contributing to stable gene silencing.

A precedent for this in relation to cell cycle exit can be found in the process of senescence. Cellular senescence is characterized by the expression of a range of cell cycle control proteins, some but not all of which are shared with plasma cells, and the reorganization of the cell nucleus with compaction of silenced chromosome domains into senescence associated heterochromatic foci (SAHF) (84). These domains do not occur in reversibly arrested cells, and are enriched for E2F target genes (84). SAHFs derive from the organization of facultative heterochromatin, associated with tri-methylated H3-K9 and tri-methylated H3-K27, into ordered structures rather than from epigenetic spreading (85–87). Thus if the organization of the plasma cell nuclear heterochromatin can be shown to resemble that of SAHFs and contain E2F target genes, it would support a process of irreversible cell cycle exit amongst such cells. If this were the case, failure to establish such highly ordered nuclear structure would be predicted amongst long-lived plasma cell that retain the potential for cell cycle entry, providing a potential mechanistic distinction between such populations.

### Gene expression changes associated with plasmablast to plasma cell transition

**Figure 1:**
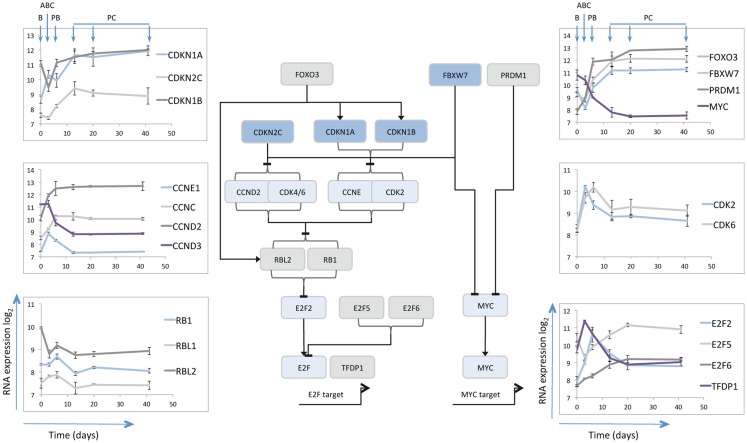
**Schematic illustration of selected cell cycle regulators**. The central line diagram provides a broad summary of relationships between the selected cell cycle regulators as identified in referenced literature. General positive (arrow heads) and negative (horizontal lines) regulatory interactions are indicated, without specific reference to the nature of the regulatory mechanism. The pattern of mRNA expression of the illustrated cell cycle regulators is shown in the scatter plots as indicated, with data derived from Cocco et al. (14). The *y*-axes show log_2_ mRNA expression values, the *x*-axes time in days, the B-cell (B), Activated B-cell (ABC), Plasmablast (PB), and Plasma cell (PC) time points are illustrated for the left and right hand top panels.

One limiting factor in analyzing plasma cell differentiation has been the inability to generate and maintain quiescent mature plasma cells *in vitro*. The resolution of this problem allowed analysis of the gene expression changes associated both with the differentiation and survival of human plasma cells over time (14). Cell cycle control was amongst the largest and most coherently regulated genetic programs. As expected, genes linked to cell cycle progression, mitosis, and cytokinesis were induced in activated B-cells maintained in plasmablasts and silenced in quiescent plasma cells. Although mRNA expression is not necessarily an accurate readout of protein expression and cell cycle control, nonetheless the gene expression changes linked to the transition from proliferating plasmablast to quiescent and long-lived plasma cells provide an indication of the cell cycle state and the potential processes operating to maintain quiescence (Figure 1).

#### MYC and CDKN2C

Consistent with the proposed pattern of regulation from prior studies (52), *MYC* expression was repressed at the plasmablast to plasma cell transition. *CDKN2C* (*p18INK4C*) followed a pattern of expression consistent with its role in murine differentiation being induced initially at the activated B-cell to plasmablast transition and then further induced at the plasmablast to plasma cell transition. Interestingly over the following weeks *CDKN2C* expression appeared to decay slightly amongst *in vitro* plasma cells that maintained cell cycle quiescence, and was expressed at only very low level in bone marrow derived plasma cell populations (data not shown) (14). A similar pattern is observed in data from another analysis of plasma cell associated gene expression (http://amazonia.transcriptome.eu/) (31). The data in human plasma cells would suggest therefore that *CDKN2C* expression is not maintained.

#### CDKN1A and CDKN1B

Amongst other negative regulators of the G1/S cell cycle checkpoint *CDKN1A* (*p21CIP1/WAF1*) was initially modestly expressed in resting B-cells induced in activated B-cells maintained at slightly lower level in plasmablasts and then sustained at high level in plasma cells. *CDKN1B* (*p27KIP1*) was expressed in B-cells, repressed in activated B-cells and induced and then maintained in plasmablasts and plasma cells. These data would therefore support the contention that CDKN1B (p27KIP1) co-operates with CDKN2C (p18INK4C) in contributing to G1/S checkpoint regulation (50), but suggest that in human plasma cells CDKN1A also contributes. Given the gradual loss of *CDKN2C* following differentiation the expression patterns support the argument that CDKN1A and CDKN1B are preferentially involved in restraining cell cycle progression once quiescence is established. Additionally the interaction of these proteins with the differentiation program may extend beyond the control of cell cycle. Thus in oligodendrocyte differentiation CDKN1B promotes cell cycle exit, while CDKN1A is linked to expression of the differentiated cell program in cells which have already exited cell cycle (88).

#### E2F5 and 6

The expression patterns of *CDKN2C* and *CDKN1A/B* at the plasmablast/plasma cell transition point reflect the central theme of G1/S control in cellular differentiation and senescence programs (49, 89), with common convergence on the control of RB phosphorylation and repression of E2F transcription factors. The E2F family includes both activators and repressors of transcription, which dimerize with transcription factor DP1 (TFDP1) to bind at common consensus sequence (90). *TFDP1* is dynamically regulated showing maximal expression in activated B-cells, but sustained expression at lower levels in plasma cells. The mRNA for activatory family member *E2F2* shows a similar pattern but with maximal expression in plasmablasts, rather than activated B-cells, and is progressively repressed with sustained plasma cell longevity. In contrast the repressive E2F family members *E2F5* and *E2F6* are distinguished by maximal expression in plasma cells. Co-expression of E2F5 and E2F6 in the absence of E2F7 is observed in quiescent rather than senescent cells (91). While E2F7 can provide a link between RB and cellular senescence programs (91), *E2F*7 mRNA was not induced upon plasma cell differentiation.

E2F family members, including E2F6, show extensive overlap in binding of DNA elements across the genome (92). However the specific expression of *E2F6* mRNA in plasma cells is of interest since E2F6 has been shown to repress E2F and MYC target genes in G0 in complex with TFDP1 and polycomb group proteins (93). Indeed E2F6 can be found in combination with several distinct functional partners implicated in the regulation of chromatin state such as EZH2, BMI1, and L3MBTL2 (94–96). Each of these genes is expressed at some level in plasma cells, but *BMI1* is notable for showing a progressive increase over time. Thus the induced expression of *E2F6* upon plasmablast to plasma cell differentiation may support the maintenance of the quiescent cell state and has the potential to contribute to epigenetic repression of cell cycle genes (93, 96).

#### Cyclins

While *Cyclin-E* (*CCNE*) mRNA was induced acutely in activated B-cells and then repressed, *Cyclin-D3* (*CCND3*) decreased rapidly during the differentiation. *Cyclin-D2* (*CCND2*) mRNA by contrast increased rapidly and was maintained at stable levels in quiescent plasma cells that no longer expressed genes associated with active cell proliferation. This is similar to the expression pattern reported in murine plasma cells (50). *CDK6* showed a spike of expression in activated B-cells and plasmablasts and was maintained at low level in mature plasma cells.

The re-entry from quiescent G0 into the G1 phase of the cell cycle can be regulated by a CCNC/CDK3 complex (97). While *CDK3* expression was not detected, CDK2 also acts as a potential alternate CCNC partner (98), and its mRNA was expressed at similar levels in plasma cells as in resting B-cells. CCNC can also act in complex with CDK8 to regulate RNA-polymerase II phosphorylation (99), but *CDK8* mRNA was minimally expressed throughout the differentiation series. In human HSCs CCNC acts in control of quiescence, with overexpression of CCNC linked to reduction and loss of CCNC to enhancement of HSC quiescence (100). It is therefore notable that *CCNC* expression increased on transition from plasmablast to plasma cell state, possibly providing a mechanism along with sustained *CCND2* mRNA expression, to poise plasma cells for cell cycle re-entry.

#### TP53, RB1, and RBL1/2

TP53, RB1 and their homologs are central to pathways of cell cycle control, and both TP53 and RB dependent pathways co-operate in the robust cell cycle exit of senescence (89). CDKN1A is a core TP53 target and its transcriptional up-regulation could be indicative of a TP53 dependent pathway in plasma cells. At mRNA level *TP53* showed modest induction in activated B-cells, at the peak of proliferative response but was subsequently present at very low level.

*RB1* was expressed at modest levels up to the plasmablast stage, but decreased at mRNA level on plasma cell maturation. *RBL1* (p107) was minimally expressed throughout differentiation, while *RBL2* (p130) displayed the most sustained expression levels. Interestingly a functional co-operation between CDKN1B and RBL2 has been identified in hemopoietic cells, and in B-cells combined deficiency of these genes leads to hyper-responsiveness to mitogenic stimuli (101).

#### FBXW7 and FOXO3

Beyond genes encoding proteins that are direct controllers of cell cycle progression and exit, other genes notably induced at the plasmablast to plasma cell transition include *FBXW7* and *FOXO3* both of which play important roles in the control of HSCs (102–104). FBXW7 is a component of the SCF-ubiquitin ligase complex and is responsible for targeting several cell cycle promoting factors including MYC and CCNE for degradation (105). Thus expression of FBXW7 can provide a mechanism to actively control pro-proliferative stimuli.

The FOXO family of transcription factors, including FOXO3, is linked to control of cell cycle progression, apoptosis, and stress resistance (106). FOXO3 can drive cell cycle exit through transcriptional regulation of CDKN1B (p27) and RBL2 (p130) (107), both of which as noted above are expressed in plasma cells. Additionally FOXO3 can induce CITED2 (108), a transcriptional co-regulator, which in HSCs is implicated in cellular survival pathways (109), and is also induced at the plasmablast to plasma cell transition. FOXO3 is itself a target of the PI3K pathway via AKT or SGK1 (110, 111). PI3K signaling is necessary for earlier stages of plasma cell differentiation (112), but has not been examined in detail in differentiated plasma cells. If FOXO3 expression is linked to control of CDKN1B and RBL2 in plasma cells then activation of the PI3K/AKT pathway may provide one means of promoting cycle re-entry.

#### Combined expression pattern

The gene expression changes during human plasmablast to plasma cell transition and subsequent maintenance are consistent with the model that cell cycle exit is established through the combined action of CDKN2C (p18), CDKN1A (p21), and CDKN1B (p27) (50), with the latter two showing more consistent expression once cell cycle exit is established. The sustained expression of *RBL2* offers a potential direct co-operation with *CDKN1B* (101). In parallel expression of *E2F5* and *E2F6* provides additional regulators that can maintain G0 quiescence (90). While *FOXO3A* identifies a potential upstream control pathway for negative cell cycle regulators (106), which is known to be responsive to PI3K/AKT signaling (110), *FBXW7* expression provides the potential for degrading mediators of cell cycle progression (105). In parallel the up-regulation of *CCNC* and sustained expression of a potential CDK partner (*CDK2*) as well as *CCND2* and *CDK6* mRNAs, could comprise a latent trigger for G0/G1 re-entry (97, 98). At a population mRNA level the induction of plasma cell quiescence is therefore associated with expression of several tiers of cell cycle repressive genes, accompanied by retained expression of cell cycle activators. This suggests the potential for regulated cell cycle re-entry.

### Monoclonal gammopathy: An expected manifestation of self-renewing plasma cell populations?

Prior to the development of aggressive myeloma, the majority of patients pass through a clinically detected or retrospectively identifiable period of indolent plasma cell neoplasia (113, 114). At the earliest stages this is characterized by the presence of a paraprotein and very low percentages of phenotypically aberrant plasma cells in the bone marrow, and is known as MGUS (115). The evolution from indolent to aggressive myeloma is thought to occur with both linear and branching patterns (79, 116–118).

In the context of the prevailing paradigm of plasma cell differentiation, the irreversible nature and the requirement for cell cycle exit in order to complete differentiation should oppose malignant transformation of mature plasma cells. This presents a potential conundrum when considering the pathogenesis of plasma cell malignancies, in particular the more indolent precursor states. There are two principle models of plasma cell malignancy: the first is that neoplastic plasma cells acquire sufficient oncogenic deregulation to escape the normal process of cell cycle exit and thus have the potential to clonally expand and progress over time (78, 79); the second is that the phenotypic plasma cell population of the malignancy is supported by a population of clonally related B-cells that act as tumor progenitors to maintain the myeloma plasma cell population (119–123). Given the complexity of human disease, each of these models may pertain in at least some instances. However while B-cell populations related to the malignant plasma cell clone are widely described (124, 125), they are not universally detected (126). Clonotypic B-cells in the peripheral blood have been found to possess early but not late oncogenic events of the myeloma clone (127). Furthermore myeloma cells expressing CD138 maintain the tumor upon transplantation into immunodeficient mice (128); and these results have been extended in recent studies showing that the capacity to establish and sustain the tumor in immunodeficient mice resides in malignant plasma cells, some of which lack CD138 expression, but not in clonotypic B-cells (129–131). Overall therefore there is strong evidence that in many instances of myeloma the disease is maintained in the neoplastic plasma cell compartment.

Given that in established myeloma the disease is maintained amongst plasma cells, earlier indolent plasma cell neoplasms including MGUS are also likely to be maintained by the neoplastic plasma cells themselves. However in this context the fraction of cells in cycle is very low, as are the number of transforming oncogenic events, while the accumulation of oncogenic events associated with cell cycle deregulation occurs later in plasma cell transformation (78, 79, 115, 118).

In MGUS and early myeloma, phenotypic differentiation is not prevented and the majority of clonal plasma cells are quiescent. In the context of a model of irreversible cell cycle exit for normal plasma cells, the initiating oncogenic event in MGUS must therefore be sufficient to establish an abnormal quiescent, but not post-mitotic state, which is not observed in normal plasma cells. A unifying model in plasma cell neoplasia has proposed that a point of convergence for initiating events is aberrant expression of D-type cyclins, and hence the deregulation of the cell cycle (132). The mechanism of deregulation and the cyclin affected differs according to the nature of the underlying molecular abnormality, but the common event is aberrant D-type cyclin expression. Effects on p18INK4C and other negative regulators of D-type cyclins and G1/S phase progression are implicated in mediating the downstream effects of such deregulation in neoplastic plasma cells, but the cells in the early phases of disease are minimally proliferative (78). How D-type cyclin deregulation suffices to establish an aberrant state of quiescent but not irreversible cell cycle exit, while the capacity to fully differentiate is maintained, remains unclear. Direct transcriptional effects of CCND1 on cell cycle gene expression have been recently reported in other lineages and may contribute (133, 134), but have yet to been investigated in plasma cells.

In contrast if transit into the long-lived bone marrow plasma cell population is linked to entry into a relative quiescent state, accompanied by an intrinsic capacity for replicative self-renewal, then the initiating oncogenic event in plasma cell malignancies driving D-type cyclin expression would not be required to deregulate an entire process of irreversible cell cycle exit. Instead it would act on a differentiated and quiescent, but not post-mitotic population to reduce the threshold for cell cycle re-entry. Over time such populations would be expected to expand relative to other long-lived plasma cells leading to gradual dominance of the clone, but given a low overall rate of cell cycle re-entry would exhibit slow clonal progression. Thus a relative quiescence model would fit with the prevailing concept of plasma cell neoplasia, and would provide a simple explanation for why small neoplastic plasma cell populations arise quite frequently and only progress to myeloma at a low rate.

## Summary and Perspectives

### A replicative self-renewal model of long-lived PC maintenance

**Figure 2:**
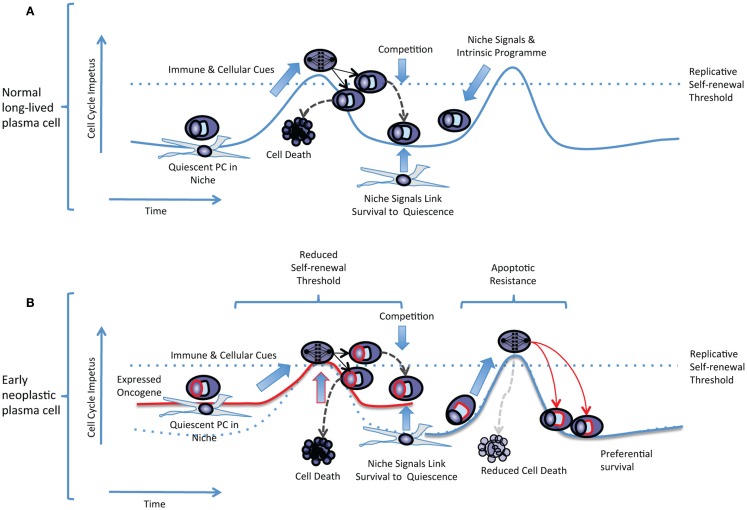
**The replicative self-renewal model of long-lived PC maintenance**. **(A)** A normal quiescent plasma cell is illustrated residing in a niche environment. The replicative self-renewal threshold is illustrated as a dotted blue line running across a hypothetical curve representing the cell cycle impetus in the quiescent plasma cell. The vertical axis represents the cell cycle impetus and the horizontal axis represents time. Niche signals and the plasma cell intrinsic program combine to establish the quiescence threshold and oppose cell cycle re-entry. Immune and cellular cues intermittently overcome the self-renewal threshold, leading to entry into cell cycle, mitosis, and generation of daughter cells that compete for survival. Successful competition for niche survival signals is linked to the re-establishment of quiescence. Failure to compete for niche occupancy leads to cell death limiting population expansion. **(B)** In an early neoplastic plasma cell expression of an oncogene such as CCND1 (illustrated as a red line surrounding the “nucleus”) reduces the effective replicative self-renewal threshold by raising the steady-state level of cell cycle impetus. Dependence on immune and cellular cues still limits cell cycle re-entry, and niche dependence contributes to the re-establishment of quiescence. In an alternate non-mutually exclusive scenario expression of an anti-apoptotic oncogene (illustrated as a red line surrounding the “secretory apparatus”) leads to preferential survival of daughter cells but the normal replicative self-renewal threshold for cell cycle entry is retained.

In a replicative self-renewal model long-lived plasma cells reside in the bone marrow niche in a quiescent state, expressing a range of cell cycle regulators, but can be triggered by cellular and immunological cues into undergoing transient episodes of replicative self-renewal at a low frequency (prevalence <1% of plasma cells) (Figure 2A). A combination of sibling-rivalry and external competition for limited niche factors would then act to constrain population expansion and link further long-term survival to the re-establishment of quiescence. For any individual antigen-specificity the frequency of plasma cell replicative self-renewal would be low, and occurring randomly would not be predicted to result in global impact on antibody titers at any single time-point. In the context of a plasma cell precursor with an expressed oncogene the threshold for cell cycle re-entry would effectively be reduced. However the plasma cell would remain constrained both by a requirement for physiological cues to trigger cell cycle re-entry, and by a dependence on niche signals for survival (Figure 2B). This would be expected to result in only gradual clonal expansion, which over time would manifest as a paraprotein and preferential clonal representation. Additional contributory mechanism, that have not been addressed here, could be envisaged from expressed oncogenes conferring apoptotic resistance leading to preferential survival of both daughter cells despite a normal self-renewal threshold. In either case the impact of the expressed oncogene would reflect a partial deregulation of a normal feature of human plasma cell biology rather than a gross perturbation of the normal differentiation process. Over time such populations would be expected to expand relative to other long-lived plasma cells. However, given a retained dependence on niche signals and capacity for establishing the normal quiescent state, would only gradually manifest as dominant clones and exhibit slow clonal progression, thus helping to explain the population of patients with MGUS who are at low overall risk of disease progression. The transition to progressive disease would be expected to arise from acquisition of additional oncogenic events eliminating the dependence on physiologic cues for replicative self-renewal, delaying re-entry into quiescence, and establishing niche independence.

### Predictions of the model

Predictions of the replicative self-renewal model would include that: (i) a population of phenotypically mature plasma cells with markers of cell cycle should be evident at low frequency in normal bone marrow, these should include cells secreting antibodies specific for prior/historic vaccine immune responses, (ii) conditions should be identifiable that promote an exit from quiescent state and re-entry of plasma cells into short bursts of re-proliferation, (iii) niche factors promoting plasma cell survival should support quiescence, and alterations in these factors should impact on cell cycle regulatory machinery as well as survival, (iv) loss of quiescence should be accompanied by cell death if quiescence is not re-established, and (v) expression of myeloma associated oncogenes should allow plasma cell differentiation and cell cycle exit but lower the threshold for cell cycle re-entry allowing enhanced clonal re-proliferation.

### Perspective

The model of relative quiescence and replicative self-renewal in long-lived plasma cells is presented here as an alternative to the currently prevailing paradigm of irreversible cell cycle exit. The latter has deep roots in plasma cell biology, and it can be argued that no direct evidence against the prevailing paradigm currently exists, but equally in the context of long-lived plasma cells how strong is the evidence in its favor? Self-renewal is an established concept amongst other memory lymphocyte populations (135, 136) and has recently been extended to tissue resident macrophages (20–22). While terminal differentiation is generally seen as a process opposing such a self-renewal capacity, quiescence with the capacity for replicative self-renewal has parallels in other cellular systems with equivalently high functional specialization (15–19). Testing these opposing models will provide a deeper understanding of the nature of cell cycle exit in plasma cells, the intersection of this process with the core plasma cell differentiation program, and the mechanism of early plasma cell neoplasia.

## Conflict of Interest Statement

The author declares that the research was conducted in the absence of any commercial or financial relationships that could be construed as a potential conflict of interest.
